# Combining the ^15^N Gas Flux Method and N_2_O Isotopocule Data for the Determination of Soil Microbial N_2_O Sources

**DOI:** 10.1002/rcm.9971

**Published:** 2024-12-26

**Authors:** Gianni Micucci, Dominika Lewicka‐Szczebak, Fotis Sgouridis, Reinhard Well, Caroline Buchen‐Tschiskale, Niall P. McNamara, Stefan Krause, Iseult Lynch, Felicity Roos, Sami Ullah

**Affiliations:** ^1^ School of Geography, Earth and Environmental Sciences University of Birmingham Birmingham UK; ^2^ Institute of Geological Sciences University of Wrocław Wrocław 50‐204 Poland; ^3^ School of Geographical Sciences University of Bristol Bristol UK; ^4^ Climate‐Smart Agriculture Thünen Institute Braunschweig Germany; ^5^ UK Centre for Ecology & Hydrology Lancaster Environment Centre Lancaster UK; ^6^ Land & Nature Division UK National Trust Swindon UK

**Keywords:** Bayesian statistics, denitrification, isotopic model, N_2_O reduction, nitrous oxide

## Abstract

**Rationale:**

The analysis of natural abundance isotopes in biogenic N_2_O molecules provides valuable insights into the nature of their precursors and their role in biogeochemical cycles. However, current methodologies (for example, the isotopocule map approach) face limitations, as they only enable the estimation of combined contributions from multiple processes at once rather than discriminating individual sources. This study aimed to overcome this challenge by developing a novel methodology for the partitioning of N_2_O sources in soil, combining natural abundance isotopes and the use of a ^15^N tracer (^15^N Gas Flux method) in parallel incubations.

**Methods:**

Laboratory incubations of an agricultural soil were conducted to optimize denitrification conditions through increased moisture and nitrate amendments, using nitrate that was either ^15^N‐labeled or unlabeled. A new linear system combined with Monte Carlo simulation was developed to determine N_2_O source contributions, and the subsequent results were compared with FRAME, a Bayesian statistical model for stable isotope analysis.

**Results:**

Our new methodology identified bacterial denitrification as the dominant process (87.6%), followed by fungal denitrification (9.4%), nitrification (1.5%), and nitrifier denitrification (1.6%). Comparisons with FRAME showed good agreement, although FRAME estimated slightly lower bacterial denitrification (80%) and higher nitrifier‐denitrification (9%) contributions.

**Conclusions:**

This approach provides an improved framework for accurately partitioning N_2_O sources, enhancing understanding of nitrogen cycling in agroecosystems, and supporting broader environmental applications.

## 1 Introduction

Nitrous oxide (N₂O) is a potent greenhouse gas, with a global warming potential 298 times greater than carbon dioxide (CO₂) over a 100‐year period [1], and which is involved in the depletion of the ozone layer [2]. Agricultural practices are a significant source of N₂O emissions, primarily due to the extensive use of nitrogen‐based fertilizers [3]. Identifying the specific microbial processes responsible for N₂O emissions is critical to better understand the underlying driving mechanisms and develop targeted mitigation strategies. Current approaches using natural abundance (NA) isotopes, such as the “isotopocule map approach” [4, 5, 6, 7], only enable to constrain the probable contributions of two sources together. Developing a model that can better discriminate individual sources would significantly enhance our understanding of N₂O field emissions.

The asymmetry of the N_2_O molecule enables us to identify N^α^, the central nitrogen atom directly adjacent to the O atom, and N^β^, the peripheral nitrogen atom of the molecule [8]. Apart from ^14^N^14^N^16^O, the three most abundant isotopocules (molecules with the same atomic identity but which differ in their isotopic composition or intramolecular position of isotopes) are ^14^N^14^N^18^O, ^14^N^15^N^16^O, and ^15^N^14^N^16^O. We can define their relative abundances as [6]

(1) $$\delta X=\frac{R_{\text{sample}}-R_{\text{standard}}}{R_{\text{standard}}}$$

where *R* is the ratio of the studied isotopocule (e.g., ^14^N^15^N^16^O in the case X = ^15^N^α^) to the most abundant one (^14^N^14^N^16^O). The standard ratios are defined by the international scale, with air N_2_ for ^15^N/^14^N and the Vienna Standard Mean Ocean Water (VSMOW) for ^18^O/^16^O. We can also define

(2) $$\delta^{15}N^{\text{bulk}}=\frac{\delta^{15}N^{\alpha }+\delta^{15}N^{\beta }}{2}$$

(3) $$\delta^{15}N^{\mathrm{SP}}=\delta^{15}N^{\alpha }-\delta^{15}N^{\beta }$$

where “bulk” refers to the average of the two most abundant ^15^N‐substituted isotopocules and “SP” to the site preference [9]. The site preference is a parameter of intramolecular distribution of the ^15^N atoms that is considered to remain constant over the course of reactions and be independent of the isotopic composition of the substrates [10], although recent studies have called this assumption into question [11]. Assuming fully equilibrated oxygen exchange between NO_3_^−^ and soil H_2_O, the δ^18^O value of N_2_O also serves as a powerful tool because the different soil microbial N_2_O sources present a relatively narrow range of δ^18^O signatures compared with δ^15^N^bulk^ [12]. Furthermore, only the determination of the δ^18^O of soil water is needed to interpret δ^18^O values, compared with all source substrates for δ^15^N^bulk^ [13]. If we consider heterotrophic nitrification, abiotic N_2_O formation, and ammonia‐oxidizing archaea contributions as negligible, then four main processes are left [4, 6, 14, 15]: bacterial denitrification (bD), fungi denitrification (fD), nitrification (Ni), and nitrifier denitrification (nD). Each of these four processes emits N_2_O with specific ranges of δ^15^N^SP^ and δ^18^O signatures available in the literature (Table 1).

**TABLE 1:** Net isotope reduction effects (ε) and isotopic signatures of the different soil N_2_O production processes.

Bacterial denitrification		Fungal denitrification		Nitrification		Nitrifier denitrification		Reduction isotopic effect	
δ^15^N^SP^ bD (‰)	δ^18^O_N2O/H2O_ bD (‰)	δ^15^N^SP^ fD (‰)	δ^18^O_N2O/H2O_ fD (‰)	δ^15^N^SP^ Ni (‰)	δ^18^O_N2O/H2O_ Ni (‰)	δ^15^N^SP^ nD (‰)	δ^18^O_N2O/H2O_ nD (‰)	ε_N‐SP_ (‰)	ε_O_ (‰)
−1.90 ± 2.8	19.20 ± 1.65	33.50 ± 3.18	47.20 ± 3.28	35.00 ± 1.68	23.50 ± 3	−5.9 ± 3.88	16.8 ± 1.25	−5 ± 1.35	−15 ± 5

*Note:* Based on Yu et al. [6]. The standard deviations of the process isotopic signatures were calculated by assuming that the ranges reported by Yu et al. [6] represented (mean + 2 standard deviations). The δ^18^O_N2O/H2O_ data are given for δ^18^O_H2O_ = 0.

When all processes are present, the resulting δ^15^N^SP^ and δ^18^O signatures of the emitted N_2_O will depend on the relative proportions of these four processes (f_bD_ + f_fD_ + f_Ni_ + f_nD_ = 1). The reduction of N_2_O to N_2_ is also associated with a shift in the isotopic signatures of δ^15^N^bulk^, δ^15^N^SP^, and δ^18^O of the residual N_2_O [16]. Classically, two scenarios are differentiated [4]. In the first one (Reduction/Mixing, “RM”), N_2_O derived from bacterial denitrification is firstly reduced into N_2_ by bacteria and then mixes with the other sources of N_2_O. In the second scenario (Mixing/Reduction, “MR”), N_2_O emitted from bacterial denitrification mixes with the other sources of N_2_O, and the mix is then reduced by bacteria. A combination of both can also theoretically occur. The RM scenario is usually considered more realistic as denitrification typically occurs in anaerobic microsites and N_2_O is likely to be reduced under these conditions [6, 12, 17].

Under the closed‐system assumption (which considers unilateral reaction and does not take into account further production of new N_2_O [18]), the shift in isotopic signature due to reduction can be approximated by [19]

(4) $$\delta_{r}-\delta_{0}\approx \varepsilon \times \ln\left(r_{N2O}\right)$$

where *δ*_0_ (‰) is the isotopic signature (either δ^15^N^bulk^, δ^15^N^SP^, or δ^18^O) of the produced N_2_O before reduction, *δ*_*r*_ (‰) is the isotopic signature of the residual unreduced N_2_O, *ε* (‰) is the net reduction isotopic effect (respectively, associated with either δ^15^N^bulk^, δ^15^N^SP^, or δ^18^O; Table 1), and *r*_*N*2*O*_ are the microscopic product ratio, which in case of the RM scenario is defined as

(5) $$r_{N2O\_\mathrm{RM}}=\frac{{\left[N_{2}O\right]}_{\mathrm{bD}}}{{\left[N_{2}O\right]}_{\mathrm{bD}}+{\left[N_{2}\right]}_{\mathrm{bD}}}$$

and in the case of the MR scenario as

(6) $$r_{N2O\_\mathrm{MR}}=\frac{{\left[N_{2}O\right]}_{\text{emitted}}}{{\left[N_{2}O\right]}_{\text{emitted}}+{\left[N_{2}\right]}_{\text{emitted}}}$$

Progress in isotopocule study has enabled the development of dual isotope plots (“isotopocule map”) for the simultaneous partition of N_2_O sources and determination of *r*_*N*2*O*_. They consist of a two‐dimensional plot underlining the relationship between two isotopic parameters, usually either δ^15^N^SP^/δ^15^N^bulk^ (SP/N map [20, 21]) or δ^15^N^SP^/δ^18^O (SP/O map [4, 5, 22]). Recently, a more precise 3‐dimensional plot encompassing δ^15^N^SP^, δ^18^O, and δ^15^N^bulk^ was developed [13]. As mentioned previously, although a powerful and useful tool, the isotopocule map approach only enables to constrain probable dominance of specific pathways together and cannot discriminate individual contributions or the relative contributions of bacteria and fungi to denitrified N_2_O emissions.

A direct method for the quantification of denitrification is the ^15^N Gas Flux method (^15^NGF), which consists of applying a ^15^NO_3_^−^ tracer to an incubated soil and measuring the abundance of ^15^N atoms in both denitrified N_2_ and N_2_O molecules [23, 24]. The resulting quantification of denitrified N_2_O and N_2_ emissions enables to derive the source partitioning coefficient (SPC) and the macroscopic product ratio (*R*_*N*2*O*_), defined as

(7) $$\mathrm{SPC}=\frac{{\left[N_{2}O\right]}_{\text{denitrified}}}{{\left[N_{2}O\right]}_{\text{emitted}}}=\frac{{\left[N_{2}O\right]}_{\mathrm{bD}}+{\left[N_{2}O\right]}_{\mathrm{fD}}}{{\left[N_{2}O\right]}_{\text{emitted}}}=f_{\mathrm{bD}}+f_{\mathrm{fD}}$$

(8) $$R_{N2O}=\frac{{\left[N_{2}O\right]}_{\text{denitrified}}}{{\left[N_{2}O\right]}_{\text{denitrified}}+{\left[N_{2}\right]}_{\text{denitrified}}}=\frac{{\left[N_{2}O\right]}_{\mathrm{bD}}+{\left[N_{2}O\right]}_{\mathrm{fD}}}{{\left[N_{2}O\right]}_{\mathrm{bD}}+{\left[N_{2}O\right]}_{\mathrm{fD}}+{\left[N_{2}\right]}_{\mathrm{bD}}}$$

The macroscopic product ratio *R*_*N*2*O*_ encompasses fungal N_2_O emissions but not fungal N_2_ emissions because fungi lack the N_2_O reductase enzyme [25]. Although more disruptive than the study of isotopocules, the ^15^NGF is generally considered more reliable to quantify denitrification [26].

So far, the NA and ^15^NGF methods have only been compared [4, 7, 13] but never integrated together. In this study, we combined them for the first time using either a linear system or Bayesian statistics. With these new approaches, we aimed to partition the N_2_O sources of an agricultural soil using laboratory incubations, where denitrification conditions were enhanced for reference (f_bd_ + f_fd_ approaching 1) and for a stronger signal of the ^15^NGF. This approach was undertaken to verify the applicability of our new method, given the high sensitivity required for N_2_O source partitioning. If validated under conditions closer to ambient (i.e., without significant stimulation of denitrification), this approach could provide more information than the isotopic map approach and discriminate more precisely individual N_2_O source contributions. If only validated under conditions favoring denitrification, this method could still determine the fungal‐to‐bacterial contribution ratio to denitrified N_2_O emissions.

## 2 Material and Methods

### 2.1 Linear System Approach

By combining the ^15^NGF and NA approaches, we get the following systems, written as augmented matrixes:

- System (S1) for the RM scenario:
$$\begin{array}{c} \begin{array}{c} \begin{array}{c} \begin{array}{c} \begin{array}{c} \left(\begin{array}{ccccc} f_{\mathrm{bD}}\left[\delta^{18}O_{\mathrm{bD}}+\varepsilon_{O}\times \ln\left(r_{N2O}\right)\right] & f_{\mathrm{fD}}\times \delta^{18}O_{\mathrm{fD}} & f_{\mathrm{Ni}}\times \delta^{18}O_{\mathrm{Ni}} & f_{\mathrm{nD}}\times \delta^{18}O_{\mathrm{nD}} & \delta^{18}O_{\text{sample}}\\ f_{\mathrm{bd}}\left[{\delta^{15}N^{\mathrm{SP}}}_{\mathrm{bD}}+\varepsilon_{N^{\mathrm{SP}}}\times \ln\left(r_{N2O}\right)\right] & f_{\mathrm{fD}}\times \delta^{15}{N^{\mathrm{SP}}}_{\mathrm{fD}} & f_{\mathrm{Ni}}\times {\delta^{15}N^{\mathrm{SP}}}_{\mathrm{Ni}} & f_{\mathrm{nD}}\times \delta^{15}{N^{\mathrm{SP}}}_{\mathrm{nD}} & {\delta^{15}N^{\mathrm{SP}}}_{\text{sample}}\\ f_{\mathrm{bD}} & f_{\mathrm{fD}} & 0 & 0 & \mathrm{SPC}\\ f_{\mathrm{bD}} & f_{\mathrm{fD}} & f_{\mathrm{Ni}} & f_{\mathrm{nD}} & 1 \end{array}\right) \end{array} \end{array} \end{array} \end{array} \end{array}$$
- System (S2) for the MR scenario:
$$\begin{array}{c} \begin{array}{c} \left(\begin{array}{ccccc} f_{\mathrm{bD}}\times \delta^{18}O_{\mathrm{bD}} & f_{\mathrm{fD}}\times \delta^{18}O_{\mathrm{fD}} & f_{\mathrm{Ni}}\times \delta^{18}O_{\mathrm{Ni}} & f_{\mathrm{nD}}\times \delta^{18}O_{\mathrm{nD}} & \delta^{18}O_{\text{sample}}-\varepsilon_{O}\times \ln\left(r_{N2O}\right)\\ f_{\mathrm{bd}}\times {\delta^{15}N^{\mathrm{SP}}}_{\mathrm{bD}} & f_{\mathrm{fD}}\times \delta^{15}{N^{\mathrm{SP}}}_{\mathrm{fD}} & f_{\mathrm{Ni}}\times {\delta^{15}N^{\mathrm{SP}}}_{\mathrm{Ni}} & f_{\mathrm{nD}}\times \delta^{15}{N^{\mathrm{SP}}}_{\mathrm{nD}} & {\delta^{15}N^{\mathrm{SP}}}_{\text{sample}}-\varepsilon_{N^{\mathrm{SP}}}\times \ln\left(r_{N2O}\right)\\ f_{\mathrm{bD}} & f_{\mathrm{fD}} & 0 & 0 & \mathrm{SPC}\\ f_{\mathrm{bD}} & f_{\mathrm{fD}} & f_{\mathrm{Ni}} & f_{\mathrm{nD}} & 1 \end{array}\right) \end{array} \end{array}$$

which have both four equations and four unknown parameters, and thus can be solved for f_bD_, f_fD_, f_Ni_, and f_nD_. For both systems, the two first equations derive from the NA approach and express how the δ^15^N^SP^ and δ^18^O signatures of the emitted N_2_O result from the combination of the four soil sources and the subsequent partial reduction to N_2_. The third equation derives directly from the ^15^NGF (Equation 7), and the fourth equation is the normalization of N_2_O emissions. Here, not only does the ^15^NGF enable to add a new equation (the third one), but it also helps in the characterization of *r*_*N*2*O*_ (by quantifying N_2_ emissions; see Equations 5 and 6), which is used in the two first equations of the system (S).

While (S2) is a linear system and easily resolved, (S1) is non‐linear. Indeed, *r*_*N*2*O*_ in the logarithm part of the bacterial denitrification isotopic signature depends on f_bD_ (see Equation 5, where [N_2_O]_bD_ depends directly on f_bD_). We assumed here that f_bD_ ≫ f_fD_ and thus *R*_*N*2*O*_ ≃ *r*_*N*2*O*_ because agricultural soils are usually bacteria‐dominated [27]. Therefore, replacing *r*_*N*2*O*_ with the determined *R*_*N*2*O*_ (which is considered constant in the current experimental conditions) will transform (S1) into a linear system that can be more easily solved. This hypothesis is tested in Section 3 of this study. However, if unverified, the model remains applicable but would require more advanced computational tools.

Solving (S1) and (S2) directly initially yielded some negative source contributions, highlighting the sensitivity of the model to the numerous parameters (and their associated uncertainties). Therefore, we used Monte Carlo simulation and generated 100 000 values of all the model parameters (source and emitted N_2_O isotopic signatures, isotopic reduction effects, SPC, and *R*_*N*2*O*_ coefficients) normally distributed around their mean and standard deviation (model data from Table 1 and result data from Table 3); and solved the linear systems (S1) and (S2) with the conditions that all source coefficients must be positive and smaller than 1. Another condition to consider carefully is that *R*_*N*2*O*_ (or *r*_*N*2*O*_) values must remain between 0 and 1. Due to the variability of the ^15^NGF in relation to N_2_ emissions, a normal distribution of these ratios with a high standard deviation may produce values outside the [0,1] interval. All calculations were performed using the R software [28].

### 2.2 Fractionation and Mixing Evaluation (FRAME) Model

As an alternative way to combine the NA and ^15^NGF approaches, we used the FRAME model. FRAME is a stable isotope modeling tool applying Bayesian statistics to evaluate isotope mixing and fractionation simultaneously [29]. The software is equipped with a user‐friendly graphical interface (malewick.github.io/frame), which does not necessitate programming skills. This calculation tool allows simultaneous source partitioning and fractionation progress determination based on the stable isotope composition of substrates and products. The mathematical algorithm applies the Markov‐Chain Monte Carlo model to estimate the contributions of individual sources and processes as well as the probability distributions of the calculated results. The open mathematical design, featuring custom distributions of source isotopic signatures, allows for the implementation of different approaches and creative modifications of the software performance. The model was designed to incorporate up to three isotopic signatures of one molecule, which is usually applied to N_2_O studies using δ^15^N^bulk^, δ^15^N^SP^, and δ^18^O values [13]. In the present study, however, the δ^15^N^bulk^ value could not be applied due to lack of precise information on the sources (δ^15^N^bulk^ of the substrates not measured), but the parallel information from the ^15^NGF approach enabled the use of the SPC as the third dimension (Equation 7). This helped constrain the model results to provide better defined sources and N_2_O reduction progress.

The four equations of the (S1) and (S2) systems have been used for the RM and MR scenarios, respectively, and all five parameters (bD, fD, nD, Ni, and *r*_*N*2*O*_) were treated as unknowns here. One of the main benefits of the FRAME model over the linear system approach is that it does not require knowledge of rN2O and thus does not rely on denitrified N_2_ data, which can be challenging to determine using the ^15^NGF. This was solved with the Monte Carlo simulation programmed in the FRAME tool, giving a probability distribution of all the unknown values. This approach allows to use the proposed calculation method to estimate N_2_O production pathway contribution also for cases where N_2_O reduction is not known.

The following values were applied in FRAME for the SPC:

- for data file: SPC = 0.9709, which is the value representing the sum of f_bD_ and f_fD_, based on the experimental results of this study (Table 3).
- for sources file: 1 for f_bD_, 1 for f_fD_, 0 for f_nD_, and 0 f_Ni_ according to the assumptions that the SPC depends only on the contributions of f_bD_ and f_fD_.
- for fractionation file: the E for SPC is 0 because it did not undergo further fractionation.

For δ^15^N^SP^ or δ^18^O, the values chosen were the same as in the linear system approach (Tables 1 and 3).

### 2.3 Laboratory Incubations

On the 15th of September 2022, arable soil (*inceptisol*) was sampled at the FarmED site, which is an experimental agricultural station near Shipton‐under Wychwood, UK (51.869981° N, 1.581136° W). The arable field at the FarmED station has been conventionally managed for wheat or barley production for 30 years. Upon return to the laboratory, soil was sieved (< 2 mm), mixed, and stored at 4°C until further processing (< 2 weeks). The soil properties were determined according to the protocols described in the Supporting Information and can be found in Table 2. We then conducted two parallel incubations: one for the NA treatment and one for the ^15^NGF treatment. Each treatment consisted of four replicates of 150 g of soil inside 450 mL sealed jars with modified lids containing septa and under conditions favoring denitrification. To that end, soil gravimetric moisture was adjusted to 70% using deionized water (~60 mL), which resulted in soil being fully water‐saturated, with about 0.2 cm of water standing on top. Furthermore, the applied deionized water contained potassium nitrate (unlabeled KNO_3_, Merck, 0.31 g L^−1^) for the NA treatment and ^15^N‐labeled potassium nitrate (K‐^15^NO_3_, 98 at %, Merck, 0.31 g L^−1^) for the ^15^NGF treatment. The quantity of added nitrate was the same for both treatments and was optimized to target a ^15^N enrichment of 50 atom % of the soil denitrifying pool in the case of the ^15^NGF experiment (considering natural ^15^N abundance in the native nitrate pool). Gas sampling took place at times 0, 3, 6, and 24 h after closure, where 30 mL of gas were sampled from the jar's headspace into 12 mL pre‐evacuated Exetainer vials (Labco Limited, UK) and 30 mL of laboratory air were added for pressure equilibration.

**TABLE 2:** Soil properties of the studied soil (mean ± standard deviation).

NO_3_^−^ (mgN/kg) *n* = 5	NH_4_^+^ (mgN/kg) *n* = 5	Bulk density (g/cm^3^) *n* = 3	Gravimetric moisture (%) *n* = 5	WFPS (%) *n* = 5	pH (in H_2_O) *n* = 5	Clay (%) *n* = 3	Silt (%) *n* = 3	Sand (%) *n* = 3	DOC (mgC/kg) *n* = 5	TDN (mgN/kg) *n* = 5
11.77 ± 0.44	1.33 ± 0.10	1.42 ± 0.04	20.34 ± 0.18	62.20 ± 0.55	7.97	82	13	5	63.78 ± 2.56	14.98 ± 0.52

Abbreviations: DOC, dissolved organic carbon; TDN, total dissolved nitrogen.

All gas samples were analyzed for N_2_O concentration using an Agilent 7890A gas chromatograph (GC) equipped with a μECD detector, for which the precision (1σ) of five repeated standard measurements was 6 ppb. In the case of the ^15^NGF experiment, isotopic ratios of N_2_ and N_2_O were acquired using a continuous flow isotope ratio mass spectrometer (IRMS, Elementar Isoprime PrecisION; Elementar Analysensysteme GmbH, Hanau, Germany), for which the precision (1σ) was (1.5 × 10^−6^) for R29, (9.3 × 10^−6^) for R30, (3.1 × 10^−5^) for R45, and (8.2 × 10^−5^) for R46. The full procedure of these two analyses is described in Micucci et al. [24]. For the ^15^NGF, denitrified N_2_O and N_2_ emissions were calculated using the equations of Mulvaney and Boast [30] and Spott et al. [31], respectively [23, 24] (see Supporting Information). The NA isotopic signatures of N_2_O were obtained using IRMS (Delta V, Thermo Fisher Scientific, Bremen, Germany) as described in Lewicka‐Szczebak et al. [4]. The precision (1σ) for δ^15^N^bulk^, δ^15^N^SP^, and δ^18^O was 0.1‰, 0.1‰, and 0.5‰, respectively. The isotopic signatures of the emitted N_2_O were extracted from the background using a mixing model [32]:

(9) $$\delta X_{\text{emitted}}=\frac{{\left[N_{2}O\right]}_{\mathrm{mix}}\delta X_{\mathrm{mix}}-{\left[N_{2}O\right]}_{\mathrm{air}}\delta X_{\mathrm{air}}}{{\left[N_{2}O\right]}_{\text{emitted}}}$$

where [*N*_2_ *O*] is the N_2_O concentration (ppm) and X is either δ^15^N^SP^ or δ^18^O. The δX_air_ (17.43‰ and 45.33‰ for δ^15^N^SP^ or δ^18^O, respectively) were determined as the average of measurements at times 0 (prior to incubation). The δ^18^O_N2O_ values were corrected by subtracting the δ^18^O value of soil water. Because δ^18^O_H2O_ analysis of soil water was not possible, we relied on the best available estimate, using local precipitation isotopic values from a nearby monitoring station (Wallingford, UK) for the month of sampling (δ^18^O_H2O_ = −4.7‰) [33]. The 2022 mean annual δ^18^O_H2O_ value at this station was (−5.6 ± 2.6 SD) ‰.

## 3 Results and Discussion

### 3.1 Experimental Results

After 3 and 6 h of incubation, soil emissions were very low, and the resulting data were under the limits of detection for both methods. However, a large activity was observed after 24 h (N_2_O concentration > 20 ppm) and was very consistent among all replicates and between the two parallel incubations. This profile of emission is typical of a wetting effect [24]. The ^15^NGF results indicated a predominance of denitrification in the N_2_O emissions with a SPC of 97% (Table 3, calculations shown in Supporting Information).

**TABLE 3:** Results for the ^15^NGF (first row) and NA (second row) experiments.

*a*_*p* (N2O)_ (%)	*a*_*p* (N2)_	N_2_ emissions (μgN kg^−1^ h^−1^)	Total N_2_O emissions (μgN kg^−1^ h^−1^)	SPC (%)	*R*_*N*2*O*_ (%)
51.14 ± 1.46	47.08 ± 2.73	3.26 ± 0.8	2.31 ± 0.06	97.09 ± 0.67	41.57 ± 4.94

δ^15^N^SP^ (‰)	δ^18^O_N2O/H2O_ (‰)	δ^15^N^bulk^ (‰)	Total N_2_O emissions (μgN kg^−1^ h^−1^)		
4.01 ± 1.70	32.80 ± 0.21	−43.53 ± 0.53	2.28 ± 0.08		

*Note:* Results are given as (mean ± 1 SD). Where *a*_*p*_ is the ^15^N enrichment of the soil denitrifying pool after ^15^NO_3_ tracer application in the case of the ^15^NGF. This enrichment was also calculated for N_2_ with the model of Mulvaney and Boast [30, 34] because the data were significantly above the detection limit, indicating high quality and readability. Calculations for the ^15^NGF are presented in the Supporting Information.

### 3.2 Model Results

For the linear system, the Monte Carlo simulation returned around 2000 solutions for (S1) and 3000 for (S2), which enabled us to constrain the source partition of N_2_O for the RM and MR scenarios (Table 4). Using the (S1) partition in combination with the N_2_O and N_2_ fluxes (Table 3) and Equation (5), we can approximate *r*_*N*2*O*_ to 45.30% in the case of the RM scenario, which is close to *R*_*N*2*O*_ (41.57%) and validates our assumption that *r*_*N*2*O*_ ≈ *R*_*N*2*O*._ If this assumption is unverified, however, (S1) is no longer a linear system and *r*_*N*2*O*_ needs to be replaced with its expression (Equation 5), which complicates the resolution of (S1). In the case of the MR scenario, we estimated *r*_*N*2*O*_ at 42.02% (Equation 6), which was also very close to *R*_*N*2*O*_ (41.57%). There were no major differences between the RM and MR scenarios; only a small contribution of bacterial denitrification (around 2%) shifted to fungal denitrification in the RM scenario. The low success rate of the Monte Carlo simulation (~2%–3%) can be attributed to the numerous parameters and their associated uncertainty range. Indeed, with 14 parameters in this model and associated standard deviations reaching up to 65% (δ^15^N^SP^ for nitrifier denitrification, Table 1), this outcome was anticipated. It could also indicate a non‐negligible contribution of the sources we ignored (heterotrophic nitrification, abiotic N_2_O formation, and ammonia‐oxidizing archaea). Indeed, given the inherent uncertainty in the model, introducing a fifth source, even with a minimal contribution, would further exacerbate this uncertainty. The resulting N_2_O source partition was nonetheless within the expected range, with a dominance of bacterial denitrification. This is further supported by the ^15^NGF, which revealed that 97% of N_2_O emissions are derived from the nitrate pool.

**TABLE 4:** Estimated source partitioning of the emitted N_2_O (mean ± standard deviation) using the two calculations models.

Model	Scenario	Bacterial denitrification (%)	Fungal denitrification (%)	Nitrification (%)	Nitrifier denitrification (%)	*r*_*N*2*O*_
LS	RM	87.6 ± 6.2	9.4 ± 6.2	1.5 ± 1.0	1.6 ± 1.0	NA
LS	MR	89.8 ± 5.1	7.1 ± 5.1	1.5 ± 1.0	1.6 ± 1.0	NA
FRAME	RM	80.5 ± 8.2	7.4 ± 5.5	2.8 ± 2.1	9.4 ± 6.6	34.7 ± 7.4
FRAME	MR	82.1 ± 7.0	6.6 ± 5.0	3.0 ± 2.3	8.3 ± 6.5	37.6 ± 7.0

*Note:* As mentioned previously, *r*_*N*2*O*_ is not considered as a variable in the LS, but a parameter determined by experiment. The FRAME model enables to re‐estimate this parameter.

Abbreviation: LS, linear system.

**FIGURE 1:**
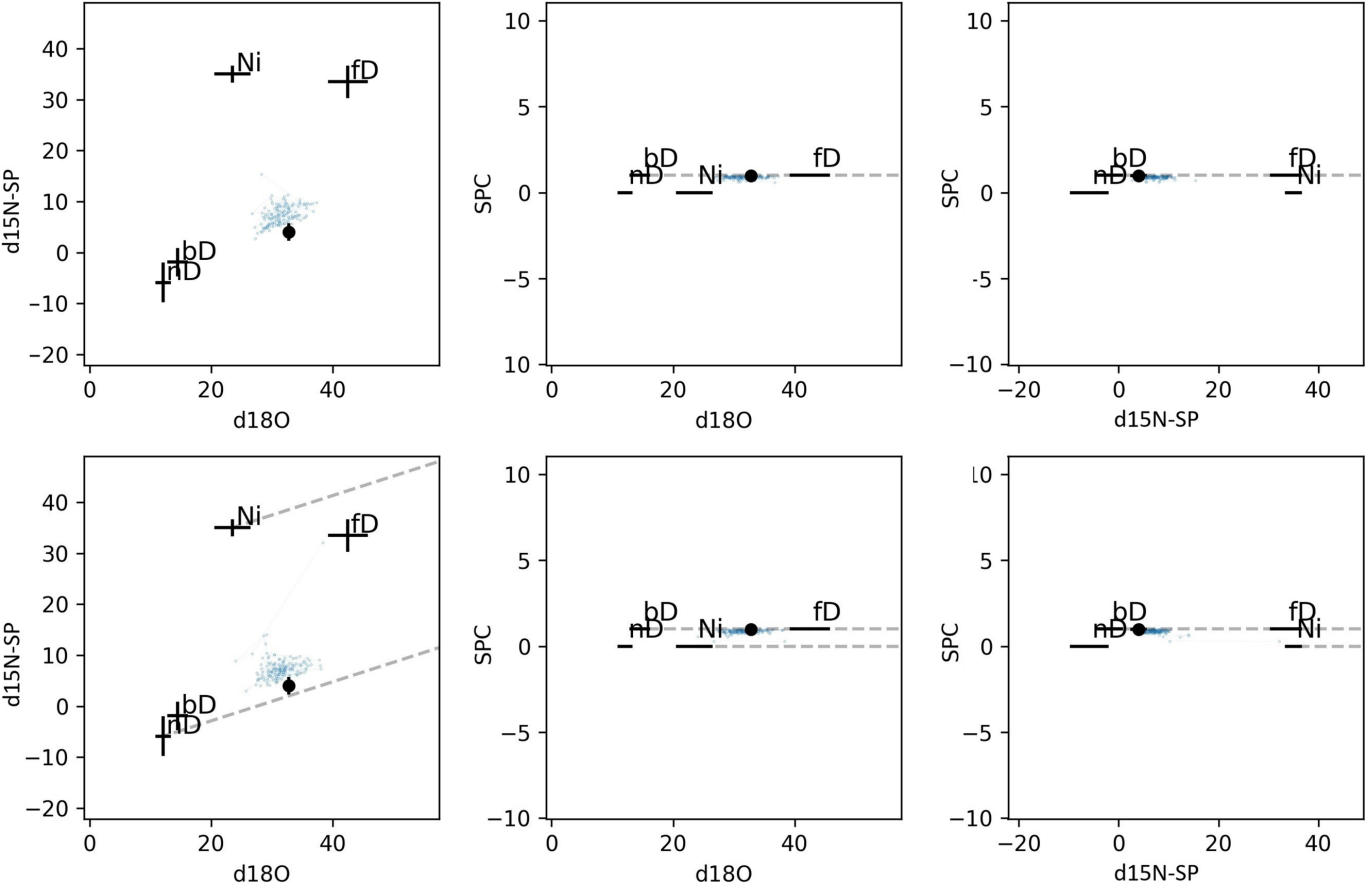
Paths of consecutive entries stored in the Markov chain plotted as two‐dimensional maps. Each blue dot represents a modeled sample value calculated for each of mixing configurations. The measurement is denoted with a black dot and error bars, while the sources with their standard deviations are represented with black crosses. Top: RM scenario, bottom: MR scenario.

The FRAME model outcomes also provided reasonable results, which were comparable to the linear approach described above, with similar ranges of uncertainties (Table 4). The model is working at the edge of possible solutions, which can be seen in Figure 1. The modeled values (blue) cannot reach the measured points (black). This is the reason why the modeling provided results only for a minority of sampling points.

**FIGURE 2:**
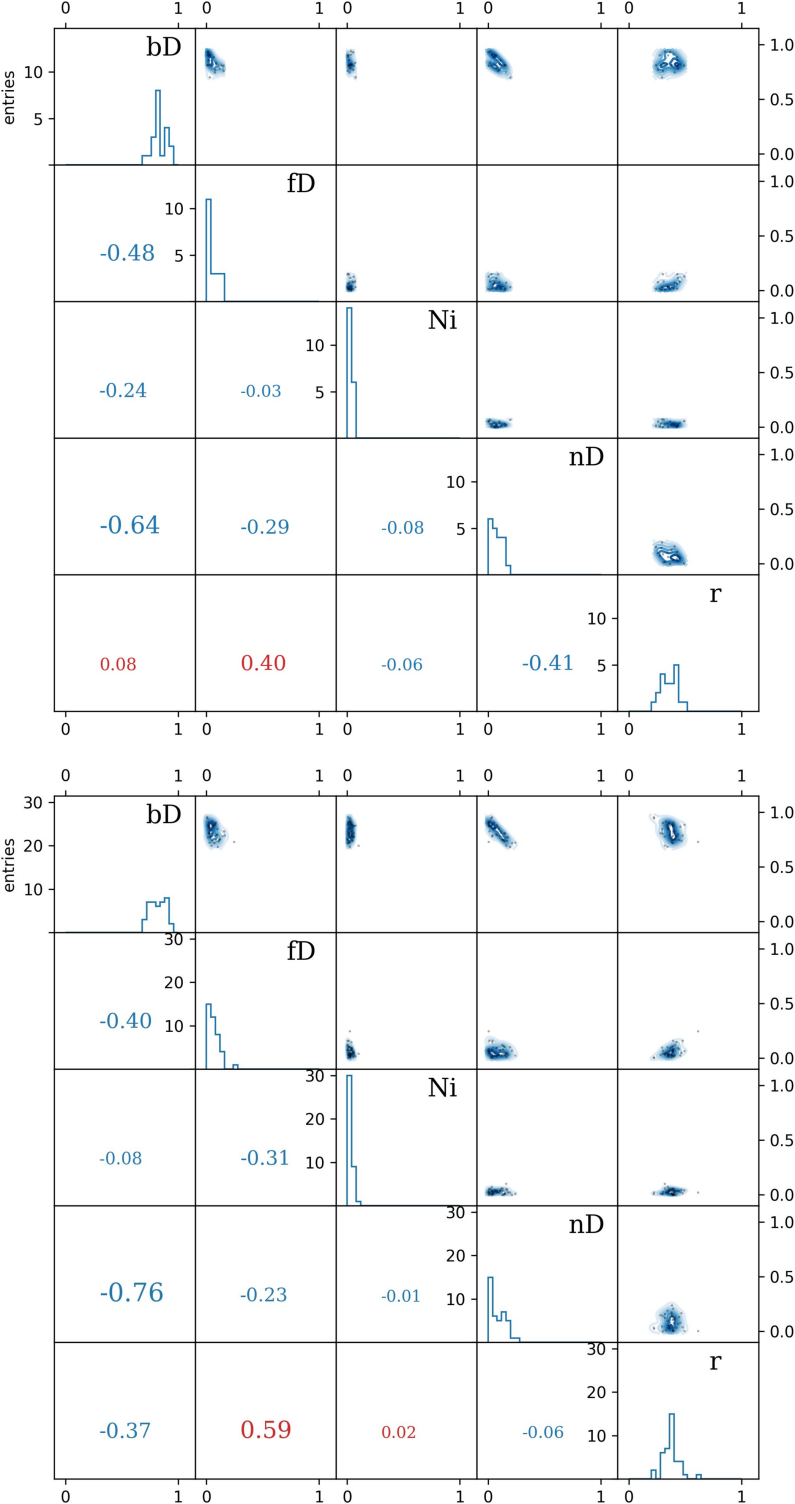
Histograms (on the diagonal) calculated from the variables building the Markov chains along with their correlations as contour‐plots (top‐right) and correlation coefficients (bottom‐left). Top: RM scenario, bottom: MR scenario.

The results of the FRAME model also indicated relatively weak correlation between nitrifier denitrification and bacterial denitrification (correlation coefficients of −0.64 for the RM scenario and −0.76 for the MR scenario, Figure 2) compared with when using only NA isotope data, where correlation coefficients are always > 0.9 (in absolute value). This indicates that the presented model is actually capable of distinguishing these two sources, which is a problem often encountered when working with NA isotopes only.

### 3.3 Model Performance and Outlook

If we compare the two approaches, the linear system found a greater contribution of bacterial denitrification (around 90% compared with 80% with FRAME). Although similar contributions of fungal denitrification were found between the two models, nitrification and nitrifier denitrification played a bigger part with the FRAME model. Indeed, we found approximately 3% and 9%, respectively, for f_Ni_ and f_nD_ with FRAME, compared with 1.5% for both when using the linear system. Similarly, FRAME enabled us to confirm that *r*_*N*2*O*_ (~35% and 38% for the RM and MR scenarios, respectively) was within the expected range, although it underestimated the values found with the linear system (~45% and 42% for the RM and MR scenarios, respectively). However, the linear system was closer to the ^15^NGF results, where *R*_*N*2*O*_ was equal to 41.57%. This can be explained by the fact that with a greater contribution of bacterial denitrification (as measured by the linear system), *r*_*N*2*O*_ becomes closer to *R*_*N*2*O*_ (Equations 5 and 8). The linear system was also more in agreement with the observed source partition. Indeed, the ^15^NGF predicted that 97% of N_2_O emissions originated from the nitrate substrate. In the present case, the two reactional pathways that use nitrate as substrate are bacterial and fungal denitrifications. We can see that the sum of f_bD_ and f_fD_ was very close to 97% with the linear system (with both the RM and MR scenarios, Table 4), where it was lower with the FRAME model (89% and 87% with the RM and MR scenarios, respectively, Table 4).

The regular isotopocule map approach from Lewicka‐Szczebak et al. [4] gives (94.78 ± 6.11)% for the sum of (f_bD_ + f_nD_) and a product ratio *r*_*N*2*O*_ of (43.34 ± 2.40)% for the RM scenario, while the MR scenario yields the same source partition and a *r*_*N*2*O*_ of (45.30 ± 4.66)%. Our results are thus generally in good accordance with this method. The linear system agrees especially well with a sum of (f_bD_ + f_nD_) close to 90% for both scenarios and a product ratio of 45% and 42% (for the RM and MR scenarios, respectively). As mentioned previously, the combination of the NA and ^15^NGF approaches enables to determine individual sources, in particular the contribution of fungal denitrification, which is not possible with the map approach. We determined with both the linear system and FRAME models that this contribution was around 8% (Table 4), while the map approach predicted a contribution of the sum (f_fD_ + f_Ni_) of 5.22%. We were also able to express a ratio of bacterial to fungal contributions to denitrified N_2_O emissions of 90%, which again cannot be derived with the classic isotopocule map approach. The determination of this ratio is possible even if the method is not sufficiently sensitive under ambient conditions by enhancing denitrification conditions, such as in the present study.

To validate and enhance our new approach for the source partition of N_2_O emissions, we recommend following several leads. First, testing our approach under conditions that do not enhance denitrification will enable to derive a realistic source partition of soil under ambient conditions and thus be more representative of field dynamics. Second, determining the δ^15^N^bulk^ signatures of the soil substrates will enable us to derive a new equation based on the δ^15^N^bulk^ of the emitted N_2_O and identical to the two first equations of (S1) and (S2). This could potentially enable the addition of an extra emission source in the model, such as heterotrophic nitrification, abiotic N_2_O formation, or ammonia‐oxidizing archaea, which were considered negligible in our approach. Similarly, a parallel incubation using ^15^N‐NH_4_^+^ (nitrification favored) could provide further information. A substrate‐induced respiration with selective inhibition (SIRIN) [34] approach could enable to block certain N_2_O production pathways and could be very useful to assess our approach. Furthermore, we flooded the soils in our experiments, which may have resulted in the accumulation of dissolved N_2_O and N_2_. Because these two gases have a different solubility in water, it may have affected their headspace concentration. Therefore, if enhancing denitrification for a higher resolution, we recommend using alternative approaches such as the addition of labile carbon sources and/or lowering the atmospheric O_2_ concentration. Finally, we did not observe major discrepancies between the RM and MR scenarios in our case, but our model could be upgraded by adding a new parameter, which would vary between 0 and 1 and would allow to have a mix of both RM and MR scenarios. This parameter could be optimized in FRAME, which would enable a more holistic model.

## 4 Conclusion

The newly developed approach allowed for the precise identification of the primary sources of soil N_2_O emissions. To our knowledge, this approach has not been attempted in the past. The present experimental conditions strongly favored denitrification, which resulted in high consistency and resolution, and enabled us to verify our methodology. Such a protocol needs to be validated under more ambient conditions to accurately represent field dynamics. However, enhancing denitrification still allows for consistent discrimination between bacterial and fungal contributions to denitrified N_2_O emissions, which has the potential to become a routine test in the future. Further work will be needed to fully evaluate this approach by applying it alongside independent validation methods.

## Author Contributions

**Gianni Micucci:** conceptualization, investigation, writing – original draft, methodology, validation, visualization, writing – review and editing, software, formal analysis, project administration, data curation. **Dominika Lewicka‐Szczebak:** validation, writing – review and editing, software, data curation, supervision. **Fotis Sgouridis:** validation, resources, supervision, writing – review and editing. **Reinhard Well:** validation, writing – review and editing, formal analysis, supervision, resources. **Caroline Buchen‐Tschiskale:** writing – review and editing, resources, supervision, formal analysis, validation. **Niall P. McNamara:** writing – review and editing. **Stefan Krause:** writing – review and editing. **Iseult Lynch:** writing – review and editing. **Felicity Roos:** writing – review and editing. **Sami Ullah:** writing – review and editing, supervision, resources, funding acquisition, project administration.

## Supporting information


**Data S1.** Supporting information.

## Data Availability

The data that support the findings of this study are openly available in Mendeley Data at https://data.mendeley.com/datasets/3jhwxfd4z3/1, reference number DOI: 10.17632/3jhwxfd4z3.1.
