# Supplementary material for: Combining the 15N Gas Flux Method and N2O Isotopocule Data for the Determination of Soil Microbial N2O Sources
Source: Rapid Commun Mass Spectrom. 2024 Dec 26;39(6):e9971. doi: 10.1002/rcm.9971 (PMC11671725; doi:10.1002/rcm.9971)
Supplement: Supplementary file 1 — Data S1. Supporting information. [file RCM-39-e9971-s001.docx]

Combining the ^15^N Gas flux method and isotopocule data for the determination of soil N_2_O sources

**Gianni Micucci**^1,*^, Dominika Lewicka-Szczebak^2^, Fotis Sgouridis^3^, Reinhard Well^4^, Caroline Buchen-Tschiskale^4^, Niall P. McNamara^5^, Stefan Krause^1^, Iseult Lynch^1^, Felicity Roos^6^ and Sami Ullah^1,7^

- 1. School of Geography, Earth and Environmental Sciences, University of Birmingham, Edgbaston, B15 2TT Birmingham, United Kingdom.
- 2. Institute of Geological Sciences, University of Wrocław, Wrocław, Poland.
- 3. School of Geographical Sciences, University of Bristol, BS8 1SS Bristol, United Kingdom.
- 4. Thünen Institute of Climate-Smart Agriculture, Bundesallee 65, 38116, Braunschweig, Germany.
- 5. UK Centre for Ecology & Hydrology, Lancaster Environment Centre, LA1 4AP Lancaster, United Kingdom.
- 6. UK National Trust, SN2 2NA Swindon, United Kingdom.
- 7. Birmingham Institute of Forest Research, University of Birmingham, Birmingham, UK.

* Corresponding author.

Email address: [giannimicucci@gmail.com](mailto:giannimicucci@gmail.com) (G. Micucci).

Supporting Information

## Soil characterization

Upon sampling, soil was sieved (< 2 mm) and placed in the fridge to be stored at 6°C. For mineral nitrogen characterization, 5 g of soil were extracted in a solution of 0.5 M Potassium Sulphate (K_2_SO_4_) at a ratio 1:8, shaken for 2 hours at 200 rpm and centrifuged at a speed of 3 000 rpm for 5 minutes. The supernatant was then filtered through a 0.45 µm syringe filter and analysed over San++ continuous flow analyser (Skalar, Breda, Netherlands). NO_3_^−^ was determine via cadmium reduction and NH_4_^+^ via the modified Berthelot reaction. The limit of detection was 0.02 mgN L^−1^ for NO_3_^−^ and 0.05 mg N L^−1^ for NH_4_^+^, the samples were blank corrected. For pH, 10 mL of deionised water were added to 5 g of soil, shaken for one hour and left to rest for another hour before measurement. Moisture was determined gravimetrically on loss of mass after 24 hours at 105°C and is reported on a per dry mass basis. Bulk density was determined by sampling a core of 250 mL, drying it at 105°C for 24 hours and weighing it. Water-filled pore space (WFPS) was determined using the bulk density and assuming a soil particle density of 2.65 g cm^-3^. For DOC and TDN quantification, 5 g of soil were extracted in 40 mL of deionised water and shaken at 200 rpm on an orbital shaker for 2 hours and filtered through n°42 Whatman paper (GE Healthcare). Filtrates were analysed over a TOC-L series (Shimadzu, Japan).

For the soil particle size, we added 5 mL of hydrogen peroxide solution (H_2_O_2_ 30 % v/v in water) to oven dried samples (24 hours at 105°C, 3 replicates per land use) at room temperature for one hour, and then again at 60°C. These operations were repeated the next day and deionised water was added (20 mL) before the samples were centrifuged (4 000 rpm for 5 minutes). The supernatant was discarded and soil particles were then dispersed into 30 mL of a sodium hexametaphosphate solution (50 g L^-1^) and analysed over a Mastersizer 2 000 (Malvern, Malvern, UK).

# Calculations of the ^15^NGF

All of these explanations are based on Micucci et al. (2023).

## N_2_ emissions:

For an IRMS with a triple collector, R29 is the ratio of ^29^N_2_ to ^28^N_2_ and R30 is the ratio of ^30^N_2_ to ^28^N_2_:

$R29=\frac{.^{29}N_{2}}{.^{28}N_{2}}$ [S1]

$R30=\frac{.^{30}N_{2}}{.^{28}N_{2}}$ [S2]

We also define *a_a_* the natural ^15^N enrichment (~0.003663), *a_p_* the ^15^N enrichment of the labelled nitrate pool, and ***d*** is the proportion of N_2_ that derives from denitrification, such as:

$d=\frac{N_{2} denitrified}{N_{2} denitrified + N_{2} atmosphere}$ [S3]

We can then determine *a_p_*, the enrichment of the labelled nitrate pool after tracer injection as:

$a_{p}=\frac{-B+\sqrt{B^{2}-4AC}}{2A}$ [S4]

where,

$A=1-2a_{a}+2\left( 1-a_{a} \right)\frac{\Delta R30}{\Delta R29}$ [S5]

$B=2{a_{a}}^{2}-2(1-{a_{a}}^{2})\frac{\Delta R30}{\Delta R29}$ [S6]

$C=2a_{a}\left( 1-a_{a} \right)\frac{\Delta R30}{\Delta R29} - {a_{a}}^{2}$ [S7]

And finally, we can determine ***d*** as:

$d=\frac{\Delta R29\left( 1-a_{a} \right)^{2}}{\frac{2(1-a_{p})(a_{p}-a_{a})}{(1-a_{a})}+\Delta R29\left( 1-a_{a} \right)^{2}-\Delta R29\left( 1-a_{p} \right)^{2}}$ [S8]

Under the headspace of an incubation vessel of constant volume, it is possible to directly express the concentration of soil-evolved N_2_ by:

$\left[ N_{2} \right]_{denitrified}=\frac{d}{1-d}\left[ N_{2} \right]_{atm}$ [S9]

Where [N_2_] is the atmospheric background (~780 000 ppm).

If the resolution is too low (especially for R30), one can re-calculate ***d*** as in Spott et al. (2006):

$d=\frac{1}{1- \frac{R29\left( 1-a_{p} \right)^{2}-2a_{p}(1-a_{p})}{R29\left( 1-a_{a} \right)^{2}-2a_{a}(1-a_{a})}}$ [S10]

Using the ***a_p_*** determined with N_2_O emissions, for which sensitivity usually much higher (see below).

## N_2_O emissions

For N_2_O, we have the following ratios:

$R45=\frac{.^{45}N_{2}O}{.^{44}N_{2}O}$ [S11]

$R46=\frac{.^{46}N_{2}O}{.^{44}N_{2}O}$ [S12]

The exact same equations as for N_2_ can be applied if doing the following corrections (based on Bergsma et al., 2001):

$R29'=R45-R17$ [S13]

$R30'=R46-\left( R29 \right)\left( R17 \right)-R18$ [S14]

where R45 and R46 are the ratios for ^45^N_2_O and ^46^N_2_O respectively, R17 is the ^17^O/^16^O ratio and R18 is the ^18^O/^16^O ratio. R17 and R18 are constant at natural abundance, Bergsma et al. (2001) used the values of 0.000373 and 0.0020052 respectively.

And similarly, one can determine:

$\left[ N_{2}O \right]_{denitrified}=\frac{d}{1-d}\left[ N_{2}O \right]_{total}$ [S15]

Where [N_2_O]_total_ is usually determined via either Gas Chromatography or Cavity Ring Down Spectroscopy.

## Calculations of SPC and R_N2O_

The two denitrification metrics can then easily be calculated as:

$SPC=\frac{{N_{2}O}_{denitrified}}{{N_{2}O}_{emitted}}$ [S16]

$R_{N2O}=\frac{{N_{2}O}_{denitrified}}{{N_{2}}_{denitrified} +{N_{2}O}_{denitrified}}$ [S17]

Where N_2_O_emitted_ is the flux of N_2_O emitted by all soil processes (usually measured via either Gas Chromatography or Cavity Ring Down Spectroscopy).

References:

Bergsma, T. T., Ostrom, N. E., Emmons, M., & Robertson, G. P. (2001). Measuring Simultaneous Fluxes from Soil of N _2_ O and N _2_ in the Field Using the ^15^ N-Gas “Nonequilibrium” Technique. *Environmental Science & Technology*, *35*(21), 4307–4312. https://doi.org/10.1021/es010885u

Micucci, G., Sgouridis, F., McNamara, N. P., Krause, S., Lynch, I., Roos, F., Well, R., & Ullah, S. (2023). The 15N-Gas flux method for quantifying denitrification in soil: Current progress and future directions. *Soil Biology and Biochemistry*, *184*, 109108. https://doi.org/10.1016/j.soilbio.2023.109108

Spott, O., Russow, R., Apelt, B., & Stange, C. F. (2006). A ^15^ N‐aided artificial atmosphere gas flow technique for online determination of soil N _2_ release using the zeolite Köstrolith SX6®. *Rapid Communications in Mass Spectrometry*, *20*(22), 3267–3274. https://doi.org/10.1002/rcm.2722
